# Supplementary material for: Statistical learning attenuates visual activity only for attended stimuli
Source: eLife. 2019 Aug 23;8:e47869. doi: 10.7554/eLife.47869 (PMC6731093; doi:10.7554/eLife.47869)
Supplement: Supplementary file 1. — Brain areas showing significant expectation suppression (GRF cluster corrected). Listed are significant clusters with their respective area label, MNI coordinate of the peak z value, the number of voxels in the cluster, as well as the p value of the cluster and its max z statistic. For large clusters (n voxels >700) additional local z maxima (z > 3.72; that is, p<0.0001, one-sided) are also shown with area label, MNI coordinates and max z statistic. Unexp. = unexpected image pairs; Exp. = expected image pairs; Att. = objects attended task; Unatt. = objects unattended (characters attended) task. [file elife-47869-supp1.docx]

## Supplementary File 1

Brain areas showing significant expectation suppression (GRF cluster corrected). Listed are significant clusters with their respective area label, MNI coordinate of the peak z value, the number of voxels in the cluster, as well as the p value of the cluster and its max z statistic. For large clusters (n voxels > 700) additional local z maxima (*z* > 3.72; i.e., *p* < 0.0001, one-sided) are also shown with area label, MNI coordinates and max z statistic. Unexp. = unexpected image pairs; Exp. = expected image pairs; Att. = objects attended task; Unatt. = objects unattended (characters attended) task.

| **Contrast** | **Area label** | **MNI coordinates** | | | **n voxels** | ***p* cluster** | **max *z*** |
| --- | --- | --- | --- | --- | --- | --- | --- |
|  |  | **x** | **y** | **z** |  |  |  |
| Unexp. > Exp. | Lateral Occipital Cortex, inferior division | -48 | -72 | -14 | 1536 | 4.1e-15 | 4.61 |
| (Att.) | Temporal Occipital Fusiform Cortex | -34 | -52 | -18 |  |  | 4.58 |
|  | Lingual Gyrus | -22 | -52 | -10 |  |  | 4.51 |
|  | Temporal Fusiform Cortex, posterior division | 30 | -38 | -20 | 535 | 3.6e-7 | 4.96 |
|  | Lateral Occipital Cortex, inferior division | 48 | -68 | -12 | 373 | 1.6e-5 | 4.24 |
|  | Precentral Gyrus | 48 | 4 | 34 | 1456 | 1.5e-14 | 4.68 |
|  | Frontal Operculum Cortex | 44 | 20 | -2 |  |  | 4.63 |
|  | Inferior Frontal Gyrus, pars opercularis | 50 | 14 | 28 |  |  | 4.56 |
|  | Frontal Orbital Cortex | 34 | 26 | -4 |  |  | 4.36 |
|  | Precentral Gyrus | -42 | -2 | 36 | 471 | 1.5e-6 | 4.36 |
|  | Frontal Operculum Cortex | -40 | 18 | 0 | 156 | 0.0079 | 3.94 |
|  | Superior Frontal Gyrus | 4 | 18 | 56 | 626 | 6.0e-8 | 4.39 |
|  | Superior Parietal Lobule | -26 | -56 | 48 | 329 | 5.0e-5 | 4.31 |
|  | Superior Parietal Lobule | 30 | -48 | 46 | 173 | 0.0046 | 4.36 |
|  | Cerebellum, Vermis VI | -4 | -64 | -18 | 128 | 0.0210 | 4.61 |
|  | Cerebellum, Left Crus I | -10 | -76 | -30 | 126 | 0.0226 | 4.32 |
|  |  |  |  |  |  |  |  |
| Unexp. > Exp. | - | - | - | - | - |  | - |
| (Unatt.) |  |  |  |  |  |  |  |
|  |  |  |  |  |  |  |  |
| [Unexp. > Exp. | Lateral Occipital Cortex, inferior division | -46 | -70 | -12 | 745 | 6.4e-9 | 4.48 |
| (Att.)] | Temporal Occipital Fusiform Cortex | -42 | -62 | -14 |  |  | 4.28 |
| > | Inferior Temporal Gyrus, temporooccipital part | -46 | -50 | -16 |  |  | 4.12 |
| [Unexp. > Exp. | Temporal Fusiform Cortex, posterior division | 30 | -38 | -24 | 173 | 0.0053 | 4.77 |
| (Unatt.)] | Lateral Occipital Cortex, inferior division | 50 | -66 | -14 | 139 | 0.0161 | 3.87 |
|  | Precentral Gyrus | 38 | 8 | 26 | 222 | 0.0012 | 4.32 |
|  | Frontal Operculum Cortex | -40 | 16 | 2 | 119 | 0.0322 | 3.81 |
|  | Lateral Occipital Cortex, superior division | -22 | -62 | 36 | 117 | 0.0345 | 3.75 |
|  | Cerebellum, Left Crus II | -10 | -76 | -34 | 125 | 0.0260 | 3.83 |
|  | Precuneous Cortex | 0 | -62 | 12 | 116 | 0.0358 | 3.84 |
|  |  |  |  |  |  |  |  |
|  |  |  |  |  |  |  |  |
